# Report of the General Board for Scotland

**Published:** 1920-03-20

**Authors:** 


### LUNACY AND MENTAL DEFICIENCY.*

### Report of the General Board for Scotland.

It is to be hoped that when once more peace is really established, and when industrial and economic conditions are more stable, increasing consideration will be given to the problems connected with the care and treatment of the insane and of the mentally deficient. Various reasons might be deduced why progress in this direction has been so slight. One is the hopeless view which has been taken as to the possibility of bringing about recovery and the consequent belief that the insane are just so many derelicts who are useless or even dangerous to the community; another is to be found in the fact that those who are engaged in the treatment of insanity have not, for the most part, known how to attract the attention of the public to a matter of vital interest.

### Signs of a Change.

There are signs, however, of a change. The Report of the Board of Control for England, dealing with the year 1918, serves to indicate this. There is, for example, a reference again to the early treatment of cases, " incipient in character or of recent origin," in " general or special hospitals, mental institutions, nursing homes, or elsewhere for limited periods, say, six months, without the necessity for certification under the Lunacy Acts." A proviso is added to this that such treatment should be " carried out under the supervision of the Board "; and it does seem advisable that some such official inspection should be arranged for in order to obviate the risk that such patients might not be in suitable surroundings, or might even be neglected. Properly conducted and equipped places would welcome such inspection—provided it were carried out tactfully and so as not to upset patients by making them feel that they were under official cognisance as lunatics: for unsatisfactory establishments such visitation is imperative. In any case some change in legislation in this direction is overdue: but when it does take place it will not introduce a new factor in the treatment of mental disorders—it will legalise and extend a method already adopted.

### Research.

Another matter referred to is research: and with this may be included the suggestion that it should be " necessary that the holders of the higher medical posts in the staffs of institutions for the insane shall possess a diploma in mental diseases, obtained after a recognised course of study in the subject." Everyone who has any acquaintance with the present position in regard to psychiatry will agree that the need for research is a very urgent one; but the efforts up to the present to provide for it may be symbolised by referring to the statement in the Report that while over £4,000,000 was spent in the year ending March 31, 1918, during the same period there was expended £375 for research in mental disease. This latter figure does not include all that has been spent, but merely represents grants made; but in any case it will serve as an indication of the general attitude towards scientific matters. This is still further exemplified when one recollects the tardiness in coping with the problem of the mental defective, a delay still further augmented by the incidence of the war.

In order, however, to have research adequately carried on there are needed not only money but men and women; though if the asylum service is made sufficiently attractive there will be no difficulty about this. There is a hint of this in the Report. The Commissioners " have for some time felt that the conditions under which asylum officers work call, in many instances, for improvement not only in pay, but in better provision for rest and recreation." The nursing staff and employees are not, nowadays, voiceless as to their requirements. Organisation has been forced on them by circumstances as it has been on many other workers, and their voice has already been heard—often with no uncertain sound. The same cannot be said of the Medical Staff who are to carry on this research, and who are to obtain for themselves these diplomas. Can it honestly be said that the asylum service, generally, is one likely to attract the best type of medical men and in sufficient numbers? It is much to be doubted. Still, it is pleasant to learn that the matter is being taken into consideration in England.

### The Scottish Report.

The Scottish Report appears to have no space for the discussion of these points. It is interesting, however, to note that the great increase in the death-rate in the Scottish asylums—nearly 18 per cent. in 1918 as compared with 1917—is attributed to influenza and not to food restrictions. As to the latter, the Commissioners are of opinion that " the asylum populations probably suffered less in this respect than the ordinary community." They were better off than those in England.† It appears the decrease in the number of notified cases of insanity has been a matter for congratulation in certain quarters; but it appears from the figures published that those who thus pride themselves on the increased mental stability of the community during the War are rather relying on the greater mortality among the insane! As a matter of fact the admissions for the year were—in England and Wales " 21,765, or 2,133 more than the number recorded in 1917, but 1,463 less than in 1914." This would

* "Lunacy and Mental Deficiency." Fifth Annual Report of the Board of Control (For the year 1918). Fifth Annual Report of the General Board of Control for Scotland.

† This question of mortality among the insane has already been discussed in these columns.—Ed., The Hospital.

**Lunacy and Mental Deficiency**—(*continued*).

seem to bear out the contention that insanity has decreased: it must be remembered, however, that there were in addition between 3,000 and 4,000 patients under care in military hospitals. Many of these would in the ordinary course have been certified and would thus have raised the number of admissions: and indeed some of them are drafted from time to time into asylums when it is seen that they are not likely to benefit by remaining in the special hospitals. As well as these cases there would be, in all probability, others who might have gone to swell the number of the certified insane had not death from wounds or from disease untimely ended their career. In Scotland there has been an actual decrease; but the Commissioners wisely remark that "the circumstances under which this decrease has occurred are of so unusual and complicated a nature that comment could not usefully be made at the present time." They add that the decrease in insanity among men cannot fail to a certain extent to be associated with military service"; and they go on to say that, possibly, the "increase of the Old Age Pension has had some effect in retarding the admission of persons suffering from senile insanity of mild types into asylums." It would be interesting to know if the influenza epidemic had not considerable influence in removing a number of potential inmates, especially of the senile type, before they came to be included in the category of the officially insane.

The Mental Deficiency Act.

In regard to the Mental Deficiency Act both Reports state that, to all intents and purposes, little has been done. Local authorities have evinced no particular anxiety to avail themselves of the powers granted by the Act; but probably their time and energy have been absorbed in other ways. The English Report states that, "One of the most potent influences that has delayed action has been financial restriction": the Act limits the annual Exchequer grant towards expenses incurred by local authorities in this connection to £150,000. In these days, when we have come to think in millions, this does seem an exiguous contribution. But there will doubtless be a considerable amendment of this later. Already, however, some progress has been made in regard to the proper care and supervision of the mentally defective, and it is to be hoped that this valuable Act will not be allowed to become a dead-letter. Efficiently carried out it should do much in time to reduce the amount of prostitution and of crime, and thus prove the preliminary outlay to have been a good investment.

There are other points raised by these Reports which are well worthy of discussion, but, in the meantime, this must suffice. It will serve to indicate that the Commissioners continue to carry on their useful and strenuous work. This will still further be shown when their report in regard to the organisation and conduct of the war hospitals is made public, as it is to be hoped it will be.

